# Systematic expression analysis of the mitochondrial complex III subunits identifies UQCRC1 as biomarker in clear cell renal cell carcinoma

**DOI:** 10.18632/oncotarget.13275

**Published:** 2016-11-10

**Authors:** Jörg Ellinger, Arabella Gromes, Mirjam Poss, Maria Brüggemann, Doris Schmidt, Nadja Ellinger, Yuri Tolkach, Dimo Dietrich, Glen Kristiansen, Stefan C. Müller

**Affiliations:** ^1^ University Hospital Bonn, Department of Urology, 53105 Bonn, Germany; ^2^ University Hospital Bonn, Department of Anesthesiology and Intensive Care, 53105 Bonn, Germany; ^3^ University Hospital Bonn, Institute of Pathology, 53105 Bonn, Germany; ^4^ University Hospital Bonn, Department of Otorhinolaryngology/Head and Neck Surgery, 53105 Bonn, Germany

**Keywords:** mitochondrial complex III, UQCRFS1, UQCRC1, biomarker, renal cell carcinoma

## Abstract

Mitochondrial dysfunction is common in cancer, and the mitochondrial electron transport chain is often affected in carcinogenesis. So far, few is known about the expression of the mitochondrial complex III (ubiquinol-cytochrome c reductase complex) subunits in clear cell renal cell carcinoma (ccRCC). In this study, the NextBio database was used to determine an expression profile of the mitochondrial complex III subunits based on published microarray studies. We observed that five out of 11 subunits of the complex III were downregulated in at least three microarray studies. The decreased mRNA expression level of UQCRFS1 and UQCRC1 in ccRCC was confirmed using PCR. Low mRNA levels UQCRC1 were also correlated with a shorter period of cancer-specific and overall survival. Furthermore, UQCRFS1 and UQCRC1 were also decreased in ccRCC on the protein level as determined using Western blotting and immunohistochemistry. UQCRC1 protein expression was also lower in ccRCC than in papillary and chromophobe subtypes. Analyzing gene expression and DNA methylation in The Cancer Genome Atlas cohort revealed an inverse correlation of gene expression and DNA methylation, suggesting that DNA hypermethylation is regulating the expression of UQCRC1 and UQCRFS1. Taken together, our data implicate that dysregulated UQCRC1 and UQCRFS1 are involved in impaired mitochondrial electron transport chain function.

## INTRODUCTION

Renal tumors are among the most common malignancies: in 2016, 62,700 new cases and 14,240 deaths were estimated in 2016 in the US [1]. Thereof, renal cell carcinoma (RCC) is the most common kidney cancer, and the clear cell subtype (ccRCC) accounts for approximately 80% of renal carcinomas. Patients with localized RCC are usually treated with curative intent, whereas cure is usually not attainable in patients with metastatic RCC. Significant therapeutic improvements have been made with the introduction of targeted antiangiogenic and immune therapies, but optimal sequencing of therapeutics is unknown [2]. Biomarkers could help to provide an individualized therapy, however such a biomarker has to be discovered.

Mitochondria are gaining an increasing interest in recent years because of their role as sensors and executioners of apoptosis and their involvement in carcinogenesis [3]. Warburg hypothesized almost one century ago that mitochondrial dysfunction in tumor cells is a major cause of carcinogenesis when he discovered that tumor cells receive energy from glycolysis rather than the mitochondrial oxidative phosphorylation. RCC is characterized by a down-regulated mitochondrial activity and a reduced activity of the mitochondrial electron transport chain [4]. However, the underlying mechanism remains to be clarified. It was earlier shown that various structural proteins of the respiratory chain undergo downregulation during renal carcinogenesis [5] including the ubiquinol-cytochrome c reductase complex [6]. The ubiquinol-cytochrome c reductase complex, also called mitochondrial complex III, is the third complex in the mitochondrial electron transport chain and plays a crucial role in the synthesis of ATP. The complex III is a composed of 11 subunits which form a multisegment transmembrane protein.

Despite of evidence for an altered expression of the complex III in RCC [6], it remains unknown whether all or only some subunits of the complex are dysregulated. The aim of this study was to develop a better understanding of the mitochondrial complex III subunits expression and to determine its potential of a new biomarker. Therefore, we reviewed microarray gene expression studies to explore the expression profile of the complex III subunits, and investigated UQCRFS1 and UQCRC1 expression in detail using PCR, Western Blot and immunohistochemistry.

## RESULTS

### Identification of dysregulated complex III subunits

**Figure 1:**
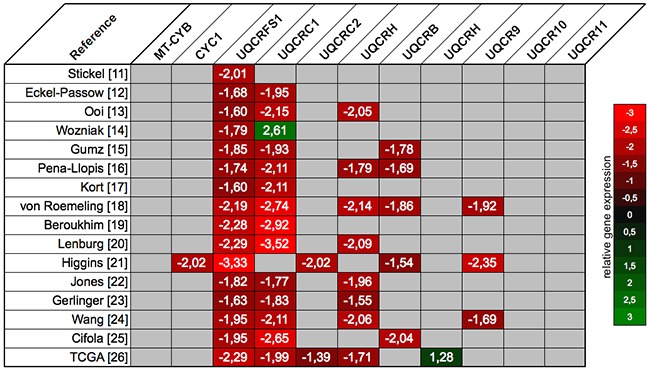
The expression profile of the mitochondrial complex III subunits was retrieved from the NextBio database: UQCRC1 and UQCRFS1 were significantly downregulated across most of the microarray gene expression studies Relative gene expression levels in ccRCC compared to normal renal tissue are scaled from red (downregulation) to green (upregulation); non-significant expression differences are coded grey.

The NextBio database included 16 different microarray studies for the comparison of normal and ccRCC tissue [7–22]. Among the 11 subunits of the mitochondrial complex III, 8 subunits were significantly dysregulated in at least one microarray study: 7 were down- and 1 was upregulated in ccRCC. However, only UQCRFS1 and UQCRC1 were significantly downregulated in all respectively 13 of 16 microarray experiments, whereas gene expression of the remaining subunits was much more inconsistent in the different studies (Figure 1).

### UQCRFS1 and UQCRC1 mRNA expression is downregulated in ccRCC

**Figure 2:**
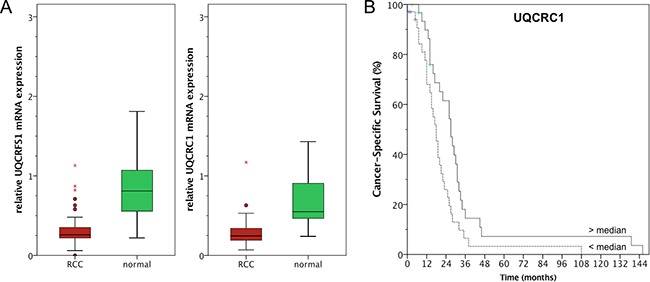
**A**. Quantitative real-time PCR confirmed lower UQCRC1 and UQCRFS1 mRNA expression in ccRCC compared to normal renal tissue. **B**. UQCRC1 levels below the median were correlated with poor cancer-specific survival (log rank p=0.013).

Gene expression of UQCRFS1 and UQCRC1 was validated using 74 ccRCC and 36 normal renal tissues. As expected, gene expression of both genes was significantly lower in ccRCC compared to normal renal tissue (both p<0.001): Mean expression UQCRFS1 levels were 0.30 (95% confidence interval 0.26-0.34) in cancer and 0.85 (0.71-0.99) in normal tissue; UQCRC1 levels were 0.28 (0.24-0.31) in cancer and 0.66 (0.56-0.75) in normal renal tissue. Thus, mRNA expression levels were decreased approximately 2.5-fold in tumor tissue (Figure 2A). Paired tumor-normal tissues were available for 19 patients; using the Wilcoxon rank test, we compared gene expression in paired tissue samples. As expected, the expression of UQCRC1 (p<0.001) and UQCRFS1 (p=0.001) was significantly reduced in the ccRCC tissue.

Both, UQCRFS1 and UQCRC1 were not correlated with clinicopathological parameters (i.e. pT-stage, pN-stage, cM-stage, grading; all p>0.05). Kaplan Meier estimates indicated a prognostic potential for both genes: Follow up information was available for 71 patients, among these 6 patients died from RCC and 2 from other causes. It should thus be noted that CSS and OS survival estimates were similar because only 3 non-RCC related deaths were observed in the cohort. UQCRC1 levels below the median were correlated with poor overall (OS; log rank p=0.015, see Figure 2B) and cancer-specific (CSS; log rank p=0.013) survival following radical/partial nephrectomy for ccRCC, whereas UQCRFS1 was not predictive of OS (log rank p=0.235) nor CSS (log rank p=0.331). Furthermore, univariate and multivariate Cox regression analysis identified UQCRC1 expression as an independent predictor of CSS and OS in ccRCC patients; see Table 1 for details. It should be noted that known risk factors like pT-stage or lymph node involvement did not predict survival and thus may reflect the limited statistical power of our cohort.

**Table 1:** Cox regression analysis for the prediction of cancer-specific and overall survival

Overall survival	univariate analysis		multivariate analysis	
	p-value	HR (95%CI)	p-value	HR (95%CI)
UQCRC1 mRNA	0.018	0.523 (0.305 - 0.897)	0.032	0.551 (0.320 - 0.949)
UQCRFS1 mRNA	0.245	0.727 (0.425 - 1.245)		
pT-stage	0.510	0.908 (0.680 - 1.212)		
LN-metastasis	0.480	2.058 (0.277 - 15.288)		
metastasis	0.062	2.161 (0.963 - 4.849)		
Grading	0.029	2.229 (1.083 - 4.587)	0.058	2.013 (0.975 - 4.155)
**Cancer-specific survival**	**univariate analysis**		**multivariate analysis**	
	**p-value**	**HR (95%CI)**	**p-value**	**HR (95%CI)**
UQCRC1 mRNA	0.016	0.522 (0.307 - 0.888)	0.027	0.548 (0.321 - 0.935)
UQCRFS1 mRNA	0.341	0.774 (0.457 - 1.312)		
pT-stage	0.563	0.920 (0693 - 1.220)		
LN-metastasis	0.501	1.989 (0.268 - 14.748)		
metastasis	0.077	2.074 (0.925 - 4.648)		
Grading	0.040	2.127 (1.036 - 4.365)	0.076	1.922 (0.934 - 3.956)

HR, hazard ratio; 95%CI, 95% confidence interval; LN, lymph node

### UQCRFS1 and UQCRC1 protein expression is decreased in ccRCC

**Figure 3:**
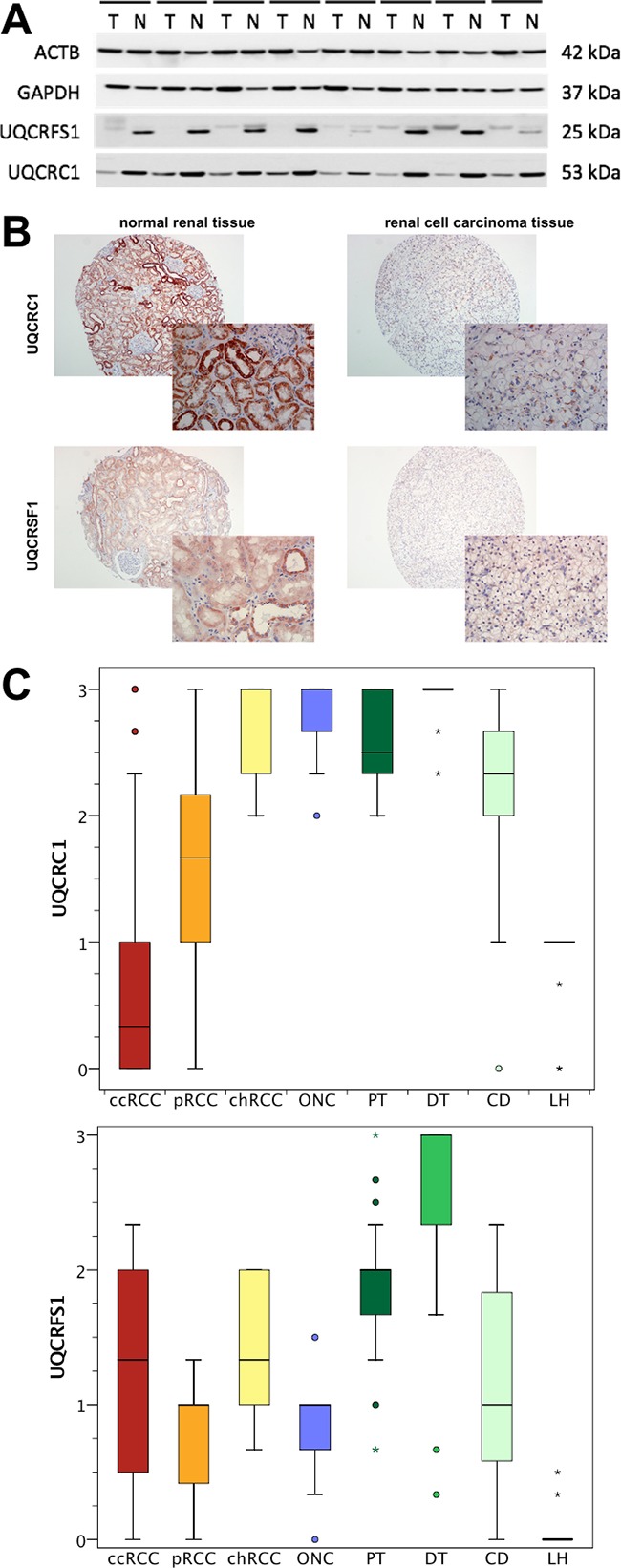
**A**. Western blot experiments were performed to determine the protein expression in 8 corresponding normal (N) and clear cell renal cell carcinoma (T) tissues. UQCRC1 and UQCRFS1 protein levels were decreased in tumor tissue. Protein levels seemed to be similar in localized (4 left) and advanced (4 right) ccRCC. **B**. The expression of UQCRC1 and UQCRFS1 was determined using immunohistochemistry in a tissue microarray. **C**. Semi-quantitative expression levels of UQCRC1 and UQCRFS1 are shown for clear cell (ccRCC), papillary (pRCC) and chromophobe (chRCC) renal cell carcinoma as well as oncocytoma (ONC) and normal renal (proximal tubules, PT; distal tubules, DT; loop of Henle, LH; collecting duct, CD) tissue. ccRCC tissues were characterized by lower UQCRC1 and UQCRFS1 levels compared to other RCC subtypes and normal renal tissue.

Next, UQCRFS1 and UQCRC1 expression was studied on the protein level using Western blot. In 8 paired normal and ccRCC tissues, both subunits were expressed at distinctly lower levels in ccRCC tissue. Expression levels seemed to be similar in tissue from patients with localized and metastatic ccRCC (Figure 3A). Interestingly, we observed a double band (approximately 25 and 28 kDa) for UQCRFS1 in most normal tissue samples; possibly, this indicates the presence of posttranslational modification (e.g. phosphorylation) or a different, so far unknown splice variant in normal renal cells.

A tissue microarray which included ccRCC (n=152), pRCC (n=29), chRCC (n=10), oncocytoma (n=10) as well as normal renal tissues (n=30) was used for immunohistochemical studies; an illustration showing staining of UQCRC1 and UQCRFS1 in normal and ccRCC tissue is provided in Figure 3B. As expected, immunohistochemical staining of UQCRC1 and UQCRFS1 was weaker in the cytoplasm of ccRCC compared to normal tissue. The in-depth analysis of the sub-compartments of the kidney, we observed significantly lower UQCRC1 (mean level: 0.62; p<0.001) and UQCRFS1 (mean levels: 1.14; p<0.001) expression in ccRCC compared to the proximal tubules (2.59; 1.82) and the distal tubules (2.97; 2.56). The number of samples with evaluable collecting duct (2.26; 1.05) and loop of Henle (0.96; 0.04) structures was low, and therefore not statistically evaluated. Notably, UQCRFS1 was almost not expressed in the loop of Henle (Figure 3C).

The tissue microarray also included samples from non-ccRCC samples. UQCRFS1 expression was somewhat lower in pRCC (mean expression level: 0.83; p=0.004), but similar in chRCC (1.40; p=0.364) and oncocytoma (0.85; p=0.0176). In contrast, UQCRC1 expression was distinctly lower in pRCC (1.63; p<0.001), chRCC (2.70; p<0.001) and oncocytoma (2.80; p<0.001).

UQCRFS1 was correlated with pT-stage (p=0.028) and Fuhrman grade (p=0.002) in ccRCC tissues. UQCRC1 was also correlated with Fuhrman grade (p=0.010). Both were not correlated with lymph node or distant metastasis (all p>0.05). The Kaplan Meier estimate indicated a non-significant trend towards poor outcome in patients with high UQCRFS1 levels, but failed to reach statistical significance (log rank p=0.093). UQCRC1 was not associated with overall survival (log rank p=0.608).

### DNA hypermethylation is associated with UQCRFS1 and UQCRC1 expression

**Figure 4:**
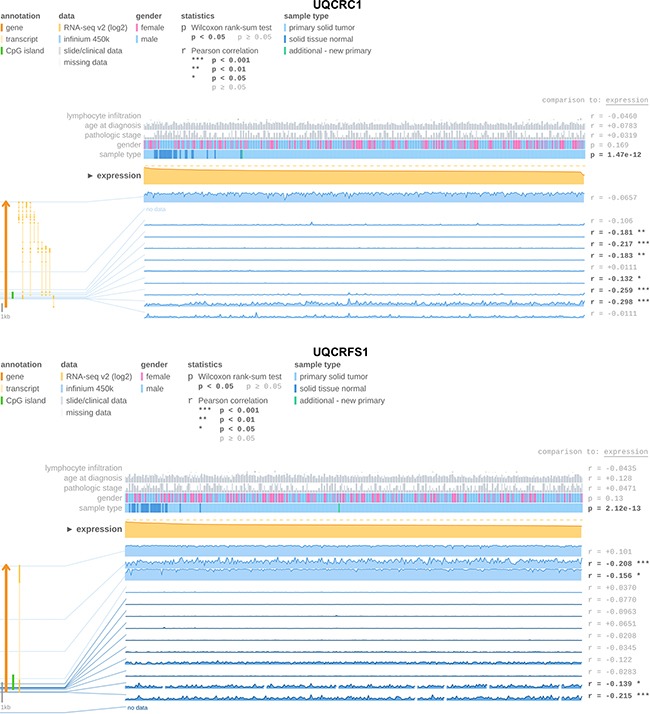
The MEXPRESS software [23] used to determine UQCRC1 and UQCRFS1 gene expression and promoter DNA methylation levels in The Cancer Genome Atlas ccRCC [22] cohort We observed an inverse expression of DNA methylation and gene expression indicating that DNA methylation is involved in gene silencing of both genes.

In order to identify potential possible causes for UQCRFS1 and UQCRC1 downregulation, we used the MEXPRESS software [23] to evaluate the relevance of DNA CpG island hypermethylation on gene expression levels in The Cancer Genome Atlas dataset [22]. DNA methylation levels of the UQCRFS1 and UQCRC promoter were inversely correlated with gene expression and methylation levels were increased in ccRCC tissue. The methylation profile of the UQCRFS1 and UQCRC promoter is shown Figure 4.

## DISCUSSION

RCC is characterized by dysregulation of multiple metabolic pathways involved in oxygen, energy and nutrient sensing cascades (deregulation of AMPK-TSC1/2-mTOR and PI3K-Akt-mTOR pathways) [24], e.g. deregulated oxygen sensing is characterized by VHL/HIF pathway alterations with subsequent upregulation of HIF-responsive genes and glucose transporters allowing the reliance on aerobic glycolysis [24]. We earlier reported dysregulation of the electron transport chain subunit NDUFA4 [25] and its paralogue NDUFA4L2 [26], and demonstrated an prognostic relevance of NDUFA4 [25]. There is evidence for an decreased expression level of the complex III in RCC [6, 27] leading to decreased activity of the electron transport chain [27]. However, former studies did not investigate whether all or only specific subunits undergo downregulation. Re-evaluation of published microarray studies revealed that only two members, UQCRC1 and UQCRFS1, were consistently downregulated in ccRCC tissue, whereas the gene expression values for the other nine subunits were contradictory or not different from normal renal tissue. This finding was confirmed experimentally for the UQCRC1 and UQCRFS1 mRNA and protein. Recently, it was shown that low expression levels of UQCRH, another ubiquinol-cytochrome c reductase complex subunit, were correlated with recurrence-free and overall survival in ccRCC patients [28].

Few is known about UQCRC1 and UQCRFS1 so far. Both genes are nuclear-encoded proteins localized at the inner mitochondrial membrane. Former studies reported deregulation of these genes in cancer: UQCRC1 expression levels were increased in osteosarcoma [29], breast and ovarian tumors [30]. The UQCRFS1 gene was overexpressed in gastric [31] and breast cancer [32–34]; gene amplification was identified as causative for upregulation in both tumors [31, 33]. Thus, UQCRC1 and UQCRFS1 seem to be involved in carcinogenesis, but deregulation may be depending upon the tumor entity, and its expression is decreased in ccRCC. Notably, downregulation of UQCRC1 and UQCRFS1 seems to be an adverse event in ccRCC, as decreased UQCRC1 mRNA expression levels were associated with a poor overall survival following nephrectomy. We observed an inverse correlation of UQCRFS1 and UQCRC1 promoter DNA methylation and mRNA levels. Similarly, UQCRFS1 was methylated in acute lymphoblastic leukemia cells [35]. The functional consequence of altered expression levels is largely unknown. Knockdown of UQCRFS1 in breast cancer cell lines reduced the mitochondrial membrane potential and impaired matrigel invasion [34].

## MATERIALS AND METHODS

### Patients

The collection of tissue samples was performed within the framework of the Biobank at the CIO Cologne-Bonn. All patients underwent radical or partial nephrectomy at the Department of Urology at the University Hospital Bonn. Written informed consent for the collection of biomaterials was obtained from all patients. The study was approved by the ethic committee of the University Bonn (vote: 280/12).

RCC and normal renal tissue was split for formalin fixation, paraffin embedding (FFPE) and fresh-frozen (FFT) storage; all tissues underwent histological examination to confirm the diagnosis. FFT were stored at -80°C, and used for qPCR (74 ccRCC and 36 normal renal tissues) and Western Blot (corresponding 8 ccRCC and normal renal tissues) experiments. FFPE tissues were used for the construction of a tissue microarray with 191 RCC specimens [comprising 152 ccRCC, 29 papillary (pRCC), 10 chromophobe (chRCC), 10 oncocytoma and 30 normal renal tissue samples were used for immunochemistry experiments [36]. All tissues were re-evaluated by an uro-pathologist, and classified according the WHO classification from 2009. The detailed clinical-pathological parameters are shown in Table 2.

**Table 2:** Clinical-pathological parameters of the study cohorts

	PCR cohort		Tissue microarray cohort				
	ccRCC	normal	ccRCC	pRCC	chRCC	oncocytoma	normal
	n=74 (%)	n=36 (%)	n=152 (%)	n=29 (%)	n=10 (%)	n=10 (%)	n=30 (%)
**Sex**							
Male	53 (71.6)	26 (62.3)	96 (62.4)	26 (89.6)	6 (60.0)	0 (0)	21 (70.0)
Female	21 (28.4)	10 (27.7)	56 (37.6)	3 (10.3)	4 (40.0)	10 (100)	9 (30.0)
**Age**							
Mean	66.5	64.8	62.2	61.5	63,2	57.6	57.9
min-max	38-83	43-89	26-85	35-82	27-85	26-73	28-80
**Pathological stage**							
pT1	42 (56.8)	n.a.	59 (38.8)	19 (65.5)	6 (60.0)	n.a.	n.a.
pT2	7 (9.5)	n.a.	33 (21.7)	5 (17.2)	4 (40.0)	n.a.	n.a.
pT3	24 (32.4)	n.a.	57 (37.5)	5 (17.2)	0 (0)	n.a.	n.a.
pT4	1 (1.3)	n.a.	3 (1.9)	0 (0)	0 (0)	n.a.	n.a.
lymph node metastasis	2 (2.6)	n.a.	13 (8.5)	1 (3.4)	0 (0)	n.a.	n.a.
distant metastasis	14 (18.9)	n.a.	24 (15.8)	3 (10.3)	0 (0)	n.a.	n.a.
**Grading**							
grade 1	10 (13.5)	n.a.	44 (28.9)	11 (37.9)	3 (30.0)	n.a.	n.a.
grade 2	46 (62.2)	n.a.	95 (62.5)	16 (55.1)	7 (70.0)	n.a.	n.a.
grade 3	15 (20.3)	n.a.	10 (6.6)	2 (6.9)	0 (0)	n.a.	n.a.
grade 4	3 (4.0)	n.a.	2 (1.3)	0 (0)	0 (0)	n.a.	n.a.

n.a., not applicable

### Quantitative real-time PCR

RNA isolation was performed as described earlier [37]. Total RNA was isolated with the mirVana miRNA Isolation Kit (Ambion, Foster City, CA, USA) and treated with DNase (Ambion). The RNA quantity was determined using a NanoDrop 2000 spectrophotometer (Thermo Scientific, Wilmington, DE, USA). The RNA integrity was checked by evaluation of the 28S and 18S rRNA bands in a gel electrophoresis.

The gene expression of two subunits, UQCRFS1 and UQCRC1, was determined using quantitative real-time PCR. cDNA was synthesized from 1 μg total RNA using the PrimeScript RT Reagent Kit with gDNA Eraser (Takara Bio, Saint-Germain-en Laye, France). PCR experiments were carried out with 5 ng/μl cDNA template, SYBR Premix Ex Taq II and ROX Plus, and 10 pmol/μl forward/reverse primer on an ABIPrism 7900 HT Fast Real-Time PCR System (Applied Biosystems, Foster City, CA, USA). Relative expression values were calculated using QBase+ (Biogazelle, Gent, Belgium) using PPIA and ACTB in the 2^-ΔΔCT^ algorithm. Primer sequences were: UQCRFS1 forward 5′-GCC-TCA-ATG-TCC-CTG-CTT-CTG-3′ and reverse 5′-CCT-AGC-CTC-GCT-GCT-TTC-TC-3′; UQCRC1 forward 5′-CAG-TCC-TCT-CAG-CCC-ACT-TG-3′ and reverse 5′-AAG-CCA-GAT-GCT-CCA-AAA-AG-3‘; ACTB and PPIA primers were published earlier [38].

### Western blot

Western blot was performed as described earlier [34] with 50 mg ccRCC and normal renal FFT. Paired samples from 8 patients (4x UICC stage I; 4x stage III) were mechanically homogenized with 400 μl cell lysis buffer (Cell Signaling, Cambridge, United Kingdom) including Complete Mini EDTA-free protease inhibitor (Roche, Basel, Switzerland). The protein concentration was determined (BCA Protein Assay Kit, Pierce Biotechnology, Rockford, IL, USA), 30 ng protein per well loaded into a NuPAGE 4-12% denaturing PAA Gel (Life Technologies, Carlsbad, CA, USA) and separated in a XCell4 SureLock electrophoresis system (Life Technologies). Biotinylated Protein Ladder (Cell Signaling Technology, Cambridge, UK) and PageRuler Prestained Protein Ladder (Thermo Scientific) were used as molecular marker. The samples were transmitted on 0.2 μm nitrocellulose (XCell II, Life Technologies) and proteins were blocked with 5 % milk powder (Merck, Darmstadt, Germany). Subsequently, immunostaining was performed with antibodies against UQCRFS1 1:1000 (#ab14746, Abcam, Cambridge, UK), UQCRC1 1:1000 (#Ab197055, Abcam), GAPDH 1:2000 (#2118, Cell Signaling Technology), and beta-actin 1:5000 (#A5316, Sigma-Aldrich). Horseradish peroxidase conjugated to secondary antibodies (anti-rabbit-POD, #170-6515, Bio-Rad Laboratories, Munich, Germany; anti-mouse-POD, #170-6516, Bio-Rad; anti-biotin-POD, #7075, Cell Signaling Technology) was used for detection. The chemiluminescent signal was visualized using SuperSignal West Femto Kit (Thermo Scientific) and recorded by the LAS 3000 Image Reader (Fujifilm, Tokyo, Japan).

### Immunohistochemistry

UQCRFS1 and UQCRC1 expression was further investigated in RCC, oncocytoma and benign renal tissue using a tissue microarray (published in[26]). Paraffin sections were cut at 5 μm thickness, deparaffinized using xylene and rehydrated in graded ethanol. Slides were placed in citrate buffer (pH 6.0) for UQCRC1 and Tris/EDTA buffer (pH 9.0) for UQCRFS1 and heated for 10 min at boiling temperature (microwave 600 W). After 30 min resting time and cooling for 15 min the endogenous peroxidase activity was blocked with 3% hydrogen peroxide for 10 minutes. The sections were washed with Tris-buffered saline and Tween 20 (Merck). The slides were incubated with the primary antibodies (UQCRFS1 1:200; UQCRC1 1:100) at 4°C overnight. Signal detection was performed with Dako Envision+ System-HRP Labeled Polymer (Dako, Hamburg, Germany) and the slides were finally counterstained using Meyers haematoxylin.

The staining was evaluated by 3 investigators (AG, MB, MP) and in case of disagreement, the scoring was discussed at a multi-headed microscope. The staining intensities were scored from 0-3, 0 being no staining to 3 being maximum staining. The expression of the target proteins was also recorded for the sub-compartments (proximal/distal tubules, collecting duct, loop of Henle) in the normal tissues.

### NextBio database

The NextBio database (Illumina, San Diego, CA, USA) was used to analyze the expression profile of the mitochondrial complex III subunits in ccRCC. The retrieval strategy for the comparison of normal renal and ccRCC tissue included the parameters “human”, “mRNA” and “fresh frozen tissue”. The last database query was performed on 20^th^ August 2015.

### Statistical analysis

Statistical analyses (t-test, Mann-Whitney-U test, Cox regression analyses, Kaplan Meier estimates) were performed, as appropriate, with SPSS Statistics v21 (IBM, Ehningen, Germany).

## References

[1] Siegel RL, Miller KD, Jemal A (2016). Cancer statistics, 2016. Cancer J Clin.

[2] Stukalin I, Alimohamed N, Heng DY (2016). Contemporary Treatment of Metastatic Renal Cell Carcinoma. Oncol Rev.

[3] Vyas S, Zaganjor E, Haigis MC (2016). Mitochondria and Cancer. Cell.

[4] Hervouet E, Godinot C (2006). Mitochondrial disorders in renal tumors. Mitochondrion.

[5] Junker H, Venz S, Zimmermann U, Thiele A, Scharf C, Walther R (2011). Stage-related alterations in renal cell carcinoma--comprehensive quantitative analysis by 2D-DIGE and protein network analysis. PLoS One.

[6] Sarto C, Marocchi A, Sanchez JC, Giannone D, Frutiger S, Golaz O, Wilkins MR, Doro G, Cappellano F, Hughes G, Hochstrasser DF, Mocarelli P (1997). Renal cell carcinoma and normal kidney protein expression. Electrophoresis.

[7] Stickel JS, Weinzierl AO, Hillen N, Drews O, Schuler MM, Hennenlotter J, Wernet D, Muller CA, Stenzl A, Rammensee HG, Stevanovic S (2009). HLA ligand profiles of primary renal cell carcinoma maintained in metastases. Cancer Immunol Immunother.

[8] Eckel-Passow JE, Serie DJ, Bot BM, Joseph RW, Hart SN, Cheville JC, Parker AS (2014). Somatic expression of ENRAGE is associated with obesity status among patients with clear cell renal cell carcinoma. Carcinogenesis.

[9] Ooi A, Wong JC, Petillo D, Roossien D, Perrier-Trudova V, Whitten D, Min BW, Tan MH, Zhang Z, Yang XJ, Zhou M, Gardie B, Molinie V (2011). An antioxidant response phenotype shared between hereditary and sporadic type 2 papillary renal cell carcinoma. Cancer Cell.

[10] Wozniak MB, Le Calvez-Kelm F, Abedi-Ardekani B, Byrnes G, Durand G, Carreira C, Michelon J, Janout V, Holcatova I, Foretova L, Brisuda A, Lesueur F, McKay J (2013). Integrative genome-wide gene expression profiling of clear cell renal cell carcinoma in Czech Republic and in the United States. PLoS One.

[11] Gumz ML, Zou H, Kreinest PA, Childs AC, Belmonte LS, LeGrand SN, Wu KJ, Luxon BA, Sinha M, Parker AS, Sun LZ, Ahlquist DA, Wood CG (2007). Secreted frizzled-related protein 1 loss contributes to tumor phenotype of clear cell renal cell carcinoma. Clin Cancer Res.

[12] Pena-Llopis S, Vega-Rubin-de-Celis S, Liao A, Leng N, Pavia-Jimenez A, Wang S, Yamasaki T, Zhrebker L, Sivanand S, Spence P, Kinch L, Hambuch T, Jain S (2012). BAP1 loss defines a new class of renal cell carcinoma. Nat Genet.

[13] Kort EJ, Farber L, Tretiakova M, Petillo D, Furge KA, Yang XJ, Cornelius A, Teh BT (2008). The E2F3-Oncomir-1 axis is activated in Wilms’ tumor. Cancer Res.

[14] von Roemeling CA, Radisky DC, Marlow LA, Cooper SJ, Grebe SK, Anastasiadis PZ, Tun HW, Copland JA (2014). Neuronal pentraxin 2 supports clear cell renal cell carcinoma by activating the AMPA-selective glutamate receptor-4. Cancer Res.

[15] Beroukhim R, Brunet JP, Di Napoli A, Mertz KD, Seeley A, Pires MM, Linhart D, Worrell RA, Moch H, Rubin MA, Sellers WR, Meyerson M, Linehan WM (2009). Patterns of gene expression and copy-number alterations in von-hippel lindau disease-associated and sporadic clear cell carcinoma of the kidney. Cancer Res.

[16] Lenburg ME, Liou LS, Gerry NP, Frampton GM, Cohen HT, Christman MF (2003). Previously unidentified changes in renal cell carcinoma gene expression identified by parametric analysis of microarray data. BMC Cancer.

[17] Higgins JP, Shinghal R, Gill H, Reese JH, Terris M, Cohen RJ, Fero M, Pollack JR, van de Rijn M, Brooks JD (2003). Gene expression patterns in renal cell carcinoma assessed by complementary DNA microarray. Am J Pathol.

[18] Jones J, Otu H, Spentzos D, Kolia S, Inan M, Beecken WD, Fellbaum C, Gu X, Joseph M, Pantuck AJ, Jonas D, Libermann TA (2005). Gene signatures of progression and metastasis in renal cell cancer. Clin Cancer Res.

[19] Gerlinger M, Horswell S, Larkin J, Rowan AJ, Salm MP, Varela I, Fisher R, McGranahan N, Matthews N, Santos CR, Martinez P, Phillimore B, Begum S (2014). Genomic architecture and evolution of clear cell renal cell carcinomas defined by multiregion sequencing. Nat Genet.

[20] Wang Y, Roche O, Yan MS, Finak G, Evans AJ, Metcalf JL, Hast BE, Hanna SC, Wondergem B, Furge KA, Irwin MS, Kim WY, Teh BT (2009). Regulation of endocytosis via the oxygen-sensing pathway. Nat Med.

[21] Cifola I, Spinelli R, Beltrame L, Peano C, Fasoli E, Ferrero S, Bosari S, Signorini S, Rocco F, Perego R, Proserpio V, Raimondo F, Mocarelli P (2008). Genome-wide screening of copy number alterations and LOH events in renal cell carcinomas and integration with gene expression profile. Mol Cancer.

[22] (2013). Comprehensive molecular characterization of clear cell renal cell carcinoma. Nature.

[23] Koch A, De Meyer T, Jeschke J, Van Criekinge W (2015). MEXPRESS: visualizing expression, DNA methylation and clinical TCGA data. BMC Genomics.

[24] Massari F, Ciccarese C, Santoni M, Brunelli M, Piva F, Modena A, Bimbatti D, Fantinel E, Santini D, Cheng L, Cascinu S, Montironi R, Tortora G (2015). Metabolic alterations in renal cell carcinoma. Cancer Treat Rev.

[25] Muller FE, Braun M, Syring I, Klumper N, Schmidt D, Perner S, Hauser S, Muller SC, Ellinger J (2015). NDUFA4 expression in clear cell renal cell carcinoma is predictive for cancer-specific survival. Am J Cancer Res.

[26] Schrodter S, Braun M, Syring I, Klumper N, Deng M, Schmidt D, Perner S, Muller SC, Ellinger J (2016). Identification of the dopamine transporter SLC6A3 as a biomarker for patients with renal cell carcinoma. Mol Cancer.

[27] Simonnet H, Alazard N, Pfeiffer K, Gallou C, Beroud C, Demont J, Bouvier R, Schagger H, Godinot C (2002). Low mitochondrial respiratory chain content correlates with tumor aggressiveness in renal cell carcinoma. Carcinogenesis.

[28] Liu WS, Liu YD, Fu Q, Zhang WJ, Xu L, Chang Y, Xu JJ (2016). Prognostic significance of ubiquinol-cytochrome c reductase hinge protein expression in patients with clear cell renal cell carcinoma. Am J Cancer Res.

[29] Liu X, Zeng B, Ma J, Wan C (2009). Comparative proteomic analysis of osteosarcoma cell and human primary cultured osteoblastic cell. Cancer Invest.

[30] Kulawiec M, Arnouk H, Desouki MM, Kazim L, Still I, Singh KK (2006). Proteomic analysis of mitochondria-to-nucleus retrograde response in human cancer. Cancer Biol Ther.

[31] Jun KH, Kim SY, Yoon JH, Song JH, Park WS (2012). Amplification of the UQCRFS1 Gene in Gastric Cancers. J Gastric Cancer.

[32] Natrajan R, Mackay A, Wilkerson PM, Lambros MB, Wetterskog D, Arnedos M, Shiu KK, Geyer FC, Langerod A, Kreike B, Reyal F, Horlings HM, van de Vijver MJ (2012). Functional characterization of the 19q12 amplicon in grade III breast cancers. Breast Cancer Res.

[33] Ohashi Y, Kaneko SJ, Cupples TE, Young SR (2004). Ubiquinol cytochrome c reductase (UQCRFS1) gene amplification in primary breast cancer core biopsy samples. Gynecol Oncol.

[34] Owens KM, Kulawiec M, Desouki MM, Vanniarajan A, Singh KK (2011). Impaired OXPHOS complex III in breast cancer. PLoS One.

[35] Nordlund J, Milani L, Lundmark A, Lonnerholm G, Syvanen AC (2012). DNA methylation analysis of bone marrow cells at diagnosis of acute lymphoblastic leukemia and at remission. PLoS One.

[36] Ellinger J, Kahl P, Mertens C, Rogenhofer S, Hauser S, Hartmann W, Bastian PJ, Buttner R, Muller SC, von Ruecker A (2010). Prognostic relevance of global histone H3 lysine 4 (H3K4) methylation in renal cell carcinoma. Int J Cancer.

[37] Blondeau JJ, Deng M, Syring I, Schrodter S, Schmidt D, Perner S, Muller SC, Ellinger J (2015). Identification of novel long non-coding RNAs in clear cell renal cell carcinoma. Clin Epigenetics.

[38] Ellinger J, Alam J, Rothenburg J, Deng M, Schmidt D, Syring I, Miersch H, Perner S, Muller SC (2015). The long non-coding RNA lnc-ZNF180-2 is a prognostic biomarker in patients with clear cell renal cell carcinoma. Am J Cancer Res.

